# Understanding osteoporosis knowledge and health beliefs in maternity nursing students: a cross-sectional study

**DOI:** 10.1371/journal.pone.0323851

**Published:** 2025-05-19

**Authors:** Faeda A. Eqtait, Bahaaeddin M. Hammad, Ahmad J. Ayed, Basma S. Salameh, Imad H. Fashafsheh, Anas H. Khalifeh, Mohammed A.L. B.ashtawy

**Affiliations:** 1 Faculty of Nursing, Arab American University, Jenin, Palestine; 2 Department of Community & Mental Health, Nursing, Faculty of Nursing, Zarqa University, Zarqa, Jordan; 3 Department of Community and Mental Health, Princess Salma Faculty of Nursing, Al al-Bayt University, Mafraq, Jordan; NUST: National University of Sciences and Technology, PAKISTAN

## Abstract

**Background:**

Osteoporosis is a growing health problem worldwide. Increasing awareness, knowledge, and promoting healthy behaviors in regard to osteoporosis and related risk factors are important targets in preventive behaviors.

**Aim:**

This study aimed to investigate maternity nursing students’ knowledge, beliefs, and preventive behaviors regarding osteoporosis.

**Methods:**

A cross-sectional study was conducted on a convenience sample of maternity nursing students at a private university in Palestine. The researchers collected data through self-administered questionnaires, included the Osteoporosis Knowledge Assessment Tool and Health Belief Scale. The collected data was analyzed using SPSS version 26, employing both descriptive and inferential statistics.

**Results:**

A total of 139 students completed the questionnaire with a response rate of 92.6%. The students demonstrated an average level of knowledge about osteoporosis with a mean score of 10.38 (SD = 4.48). Common risk factors identified were caffeine intake (69.9%), smoking (55.9%), and low calcium intake (46.3%). The mean Osteoporosis Health Belief Scale score was 124.87 (SD = 24.21), with high perceived benefits for exercise (22.43 ± 5.61) and calcium intake (21.01 ± 5.11) but low susceptibility (13.26 ± 4.77). Knowledge was significantly higher in fourth-year students (p = 0.034) and overweight participants (p = 0.020). Calcium supplementation predicted health belief scores (p = 0.011), while academic year and sun exposure had no significant impact.

**Conclusion:**

The current study confirmed that maternity nursing students had average knowledge about osteoporosis. There is a gap between the students’ beliefs about osteoporosis and their daily lifestyle. Therefore, a special attention should be paid to raise the level of osteoporosis awareness among nursing students through health education program. as well as broader integration of osteoporosis in nursing curricula is urgently warranted.

## Introduction

Osteoporosis is a major public health problem affecting millions of individuals worldwide. Osteoporosis is characterized by reduced bone mass and the deterioration of bone microarchitecture, is not a normal part of aging. These changes significantly increase the risk of fractures, particularly in the hip, spine, and wrist, leading to disability, reduced quality of life, and increased mortality rates. The socioeconomic burden of osteoporosis is substantial, with escalating healthcare costs and long-term dependence on medical care for affected individuals [1,2].

The International Osteoporosis Foundation (IOF) estimates that approximately 200 million people worldwide are affected by osteoporosis. In Europe alone, the prevalence among individuals aged 50 years and older is projected to increase by 20% by 2025, with associated healthcare costs expected to rise by 25% [3,4]. Osteoporosis is particularly prevalent in Arabic countries, where rates range from 8% to 40% [4,5]. For instance, in Jordan, prevalence rate estimates range between 13.5% and 44% [6]. In Palestine, osteoporosis affects 30% of postmenopausal women at the lumbar spine, 14% at the total hip, 24% at the femoral neck, and 41% at any site [7].

Although osteoporosis can affect both men and women, approximately 80% of affected individuals are women [8]. Fractures are the most serious complication of osteoporosis, with their incidence increasing with age. Globally, one in three women is at risk of osteoporotic fractures, and between 20% and 25% of individuals will experience a fracture in their lifetime. Among patients with osteoporosis-related hip fractures, the mortality rates during the first two years range from 12% to 20%. Additionally, nearly 50% of older adults become dependent on others after a fracture, further highlighting the crucial need for preventive measures [4,9].

Considering its significant public health impact, there has been an increasing global emphasis on osteoporosis prevention [10]. Approaches such as awareness campaigns, lifestyle modifications, and early intervention have proven effective in reducing the risk of osteoporosis-related fractures [11–14]. Therefore, educating women about osteoporosis and encouraging the adoption of a healthy lifestyle are essential to prevention efforts. However, existing literature indicates a persistent lack of knowledge and awareness about osteoporosis among women across diverse age groups, including university students. A large proportion neglect to adopt preventive behaviors like regular exercise and adequate calcium intake [15,16].

International studies reveal a general deficiency in osteoporosis awareness extending beyond the general population to include healthcare professionals [15–17]. Although various studies have assessed osteoporosis knowledge among healthcare providers, most have been conducted in Western countries [10,12,18–24], with limited research in the Arabic population [24–28]. In Palestine, studies on postmenopausal women reveal poor dietary habits, low physical activity levels, and inadequate knowledge about osteoporosis risk factors [7]. This pattern is also evident among young Arab females, including nursing students, many of whom lack adequate knowledge and maintain unhealthy behaviors [24,26,27]. However, participation in health education programs has been shown to significantly improve osteoporosis knowledge and awareness [10,28].

Despite extensive research on osteoporosis, little is known about maternity nursing students’ knowledge and health beliefs in Palestine. As future healthcare providers, maternity nursing students play an essential role in educating pregnant women about bone health, calcium and vitamin D intake, and lifestyle modifications that can prevent osteoporosis later in life. Understanding their knowledge, beliefs, and preventive behaviors is crucial for designing targeted educational interventions. Therefore, this study aims to assess osteoporosis knowledge, health beliefs, and preventive behaviors among maternity nursing students in Palestine

## Materials and methods

### Design and participants

This study employed a descriptive cross-sectional survey using self-administered questionnaires. This study was carried out among nursing students enrolled in a maternity nursing health course at a private university in Palestine. A convenience sampling technique was utilized to collect data.

### Sample size

The sample size was calculated using Raosoft® online sample size calculator at a 95% confidence interval and population size of 185 and 5% accepted margin error. The sample size included in the study was 126 students to be included in the study. We increased 10% of the calculated sample size to overcome problems in data collection so the targeted number of students for collect data from them was 139 students.

### Instruments

In this study, a set of instruments will be used to measure various variables. The first section of the questionnaire will gather demographic information including age, gender, height, weight, and academic year. Participants will also respond to questions about osteoporosis prevention behaviors and risk factors based on the literature [10,29], covering physical activity, body mass index (BMI), smoking, caffeine intake, dairy product consumption, calcium supplements, and sun exposure.

The second section will evaluate participants’ osteoporosis knowledge using the Osteoporosis Knowledge Assessment Tool (OKAT) [30]. This tool comprises 20 True/False/Do not Know items, with one point awarded for each correct answer. Incorrect and “do not know” responses will receive zero points. The maximum possible score on the OKAT is 20, with higher scores indicating greater knowledge about osteoporosis. It assesses knowledge related to the disease process, risk factors, prevention, and treatment of osteoporosis. The Arabic version of the OKAT, translated by Sayed-Hassan and Bashour [25], has a Cronbach’s alpha coefficient of 0.824.

In the third section, participants’ beliefs about osteoporosis will be measured using the Osteoporosis Health Belief Scale (OHBS) [31], based on the health belief model. The OHBS is a 42-item questionnaire designed to evaluate beliefs related to osteoporosis. It consists of seven subscales, including perceived susceptibility to osteoporosis, seriousness of osteoporosis, benefits of exercise, benefits of calcium intake, barriers to exercise, barriers to calcium intake, and health motivation. Responses are given on a 5-point Likert scale, ranging from strong disagreement (1) to strong agreement (5), resulting in a possible score range of 6–30 for each subscale and 42–210 for the total scale. For most subscales, higher scores indicate healthier beliefs, while for the two barrier subscales, higher scores suggest more negative health beliefs. The Arabic version of the OHBS, translated by Sayed-Hassan and Bashour [25], has a Cronbach’s alpha coefficient of 0.806. Scores in each health belief domain were categorized into five categories: low (6–11), moderately low (12–16), neutral (17–19), moderately high (20–24), and high (25–30) [32].

### Ethical considerations

Prior to data collection, ethical approval was obtained from the Institutional Review Board at Arab American University - Palestine (2023/A/152/N). Participants provided written informed consent after receiving detailed information about the study’s objectives, potential benefits and their write. They were assured of anonymity, confidentiality, and that participation was completely voluntary. Additionally, participants were explicitly informed of their right to withdraw from the study at any time without any consequences. The study adhered strictly to the principles delineated in the Helsinki Declaration.

### Data collection

Once ethical approval obtained, the principal researcher met with eligible nursing students in their lecture rooms to extend invitations to participate in the study. Before obtaining their consent, each student was thoroughly informed about the study’s objectives and nature. Participants who agree to take part in the study was provide with a questionnaire package, accompany by a cover letter that explains the purpose of the research. The students were asked to complete the entire set of questionnaires and return them to the researcher. Returning the completed questionnaires were considered an implied consent for participation in the study. Data collection took place between October, 2023 to January, 2024.

### Data analysis

The data analysis included descriptive and inferential statistics. Descriptive statistics (mean, standard deviation, frequency, and percentage) summarized demographics characteristics, osteoporosis knowledge, and health beliefs. Independent t-tests and one-way ANOVA compared osteoporosis knowledge and OHBS scores across demographic groups. Pearson’s correlation assessed relationships between osteoporosis knowledge, health beliefs, and lifestyle factors. While multiple regression examined predictors of knowledge and health beliefs. Analyses were conducted using SPSS version 26, with significance set at p < 0.05.

## Results

### Description of sample characteristics

Out of 150 distributed questionnaires, 139 were returned, yielding a 92.6% response rate. After excluding three incomplete questionnaires, the final sample included 136 participants. The majority were male (51.5%, n = 70) and in their third year of study (61.8%, n = 84). Most participant (97.1%, n = 132) reported had knowledge about osteoporosis, primarily acquired through university courses (50%, n = 68). The mean age was 20.92 years (SD = 0.74) and a mean BMI of 22.07 (SD = 1.99).

Regarding osteoporosis risk factors, “inadequate intake of calcium-rich nutrients” was the most commonly reported (46.3%, n = 63), followed by “caffeine intake” (69.9%, n = 95) and “smoking” (55.9%, n = 76). Less frequently reported risk factors included “avoiding sun exposure” (14%, n = 19), additionally, 13.2% (n = 18) of participant were classified as overweight based on BMI, as presented in Table 1.

**Table 1:** Demographic characteristics of the participating students (N = 136).

Variable		% (n)
**General Demographics**		
Age, Mean (SD)		20.92 (0.74)
Body Mass index, Mean (SD)		22.07 (1.99)
Category of Body Mass index		
	Under weight	2.2 (3)
	Ideal weight	84.6 (115)
	Over weight	13.2 (18)
Gender		
	Male	51.5 (70)
	Female	48.5 (66)
Academic Year		
	Third	61.8 (84)
	Fourth	38.2 (52)
**Osteoporosis Knowledge**		
Knowledge about Osteoporosis		
	Yes	97.1 (132)
	No	2.9 (4)
Source of Knowledge		
	Clinical training	11.8 (16)
	Theory courses	50 (68)
	Social media	38.2 (52)
**Lifestyle and Health related factors**		
Smoking		
	Yes	55.9 (76)
	No	44.1 (60)
Caffeine intake		
	Yes	69.9 (95)
	No	30.1 (41)
Doing regular exercise		
	Yes	46.3 (63)
	No	53.7 (73)
Intake nutrient rich in calcium		
	Inadequate	46.3 (63)
	Moderate inadequate	33 (24.3)
	Adequate	40 (29.4)
Taking Calcium supplement		
	Yes	19.1 (19.1)
	No	80.9 (110)
Exposure to sun		
	Yes	86 (117)
	No	14 (19)

### Knowledge of osteoporosis

The findings indicate that maternity nursing students had an average level of osteoporosis knowledge, with a mean OKAT score of 10.38 (SD = 4.48) out of 20 points, representing 57.4% of the maximum achievable score. Detailed knowledge items scores are provided in Table in “S1 Table”. The highest scoring item was item 1, “Osteoporosis leads to an increased risk of bone fractures,” with 94.1% of participant answering correctly, followed by item 11“It is easy to tell whether I am at risk of osteoporosis by my clinical risk factors” at 76.5%. In contrast, the lowest scoring items were items 2 “Osteoporosis usually causes symptoms before fracture occur” with only 5.9% of participants answering correctly and item 18 “There is a small amount of bone loss in the ten years following the onset of menopause” at 19.1%.

### Health beliefs towards osteoporosis

The findings reveal that the mean OHBS score across all items was 124.87 (SD = 24.21, range 42–171), representing 64.4% of the maximum possible score, as presented in Table 2. Regarding the seven OHBS dimensions, participants scored moderately low in perceived barrier to exercise (14.32 ± 4.55) and susceptibility to osteoporosis (13.26 ± 4.77). They held neutral beliefs about the seriousness of osteoporosis (17.73 ± 5.23) and barrier to calcium intake (17.72 ± 4.51). While they demonstrated moderately high scores for perceived benefits of exercise (22.43 ± 5.61), health motivation (21.40 ± 5.56) and perceived benefits of calcium intake (21.01 ± 5.11).

**Table 2:** The mean scores of OHBS dimension among maternity nursing students.

Variable	Mean	SD	Minimum	Maximum
Osteoporosis health beliefs, total score	124.87	24.21	42	171
Susceptibility of osteoporosis	13.26	4.77	6	30
Seriousness of osteoporosis	17.73	5.23	6	30
Benefits of exercises	22.43	5.61	6	30
Benefits of calcium intake	21.01	5.11	6	30
Barriers to exercises	14.32	4.55	6	29
Barriers to calcium intake	17.72	4.51	6	28
Health motivation	21.40	5.56	6	30

SD, Standard Deviation

### Comparison of study variables based on participants’ demographics

The analysis revealed no significant difference in total OHBS scores between gender (p = 0.58) or in knowledge scores between gender (p = 0.65), suggesting that gender does not significantly influence osteoporosis related health believe or knowledge. Regarding academic year, no significant difference was found in total OHBS scores between third- and fourth-year students (p = 0.51). however, a significant difference was observed in knowledge scores, with fourth-year students showing significantly higher scores compared to third year students (p = 0.034). Exposure to osteoporosis-related information did not significantly influence total OHBS mean score (p = 0.28) or knowledge score (p = 0.36).

Furthermore, The ANOVA result indicated no significant differences in total OHBS score across BMI categories (p = 0.65). While a significant difference was found in knowledge scores (p = 0.020), with overweight participants showing significantly higher knowledge scores than those in the ideal weight category (p = 0.034). Finally, no significant differences were found in total OHBS (p = 0.523) or knowledge scores (p = 0.372) across different calcium intake categories. Similarly, no significant differences were observed in total OHBS (p = 0.64) or knowledge scores (p = 0.18) across different sources of osteoporosis-related information, as presented in Table 3.

**Table 3:** Comparison of the study variables based on Participants’ Demographics (N = 136).

Variable		Knowledge mean Score		OHBS mean score	
		Mean (SD)	P-value	Mean (SD)	P-value
Gender			0.65		0.58
	Male	10.54 (4.92)		123.74 (23.07)	
	Female	10.20 (3.98)		126.06 (25.48)	
Academic Year			0.034		0.51
	Third	9.74 (4.26)		123.79 (22.96)	
	Fourth	11.40 (4.66)		126.62 (26.23)	
Knowledge about Osteoporosis			0.28		0.36
	Yes	10.45 (4.35)		124.54 (24.41)	
	No	8 (8.25)		135.75 (13.77)	
Intake nutrient rich in calcium			0.37		0.52
	Inadequate	9.87 (5.07)		122.9 (27.77)	
	Moderate inadequate	10.39 (3.53)		128.85 (19.93)	
	Adequate	11.15 (4.15)		124.68 (21.37)	
Category of Body Mass index			0.02		0.65
	Under weight	13.33 (5.03)		127.33 (29.02)	
	Ideal weight	9.92 (4.32)		124.05 (24.9)	
	Over weight	12.78 (4.66)		129.67 (19.17)	
Source of Knowledge			0.18		0.64
	Clinical training	9.31 (4.73)		119.75 (21.60)	
	Theory courses	11.07 (4.38)		126.15 (21.72)	
	Social media	9.79 (4.46)		124.77 (21.92)	

SD, Standard Deviation

The correlation analysis identified several key relationships between osteoporosis knowledge, health belief scores, student demographics, and lifestyle and health-related factors. For osteoporosis knowledge, a significant positive correlation with academic year (r = 0.181, p = 0.034) was observed, suggesting that students’ knowledge of osteoporosis increases as they advance in their academic studies. A weak, non-significant positive trend was found between osteoporosis knowledge and exposure to sun (r = 0.137, p = 0.111) and nutrient-rich calcium intake (r = 0.121, p = 0.161), indicating potential associations between sun exposure, dietary habits, and osteoporosis awareness. However, no significant relationships were identified between osteoporosis knowledge and caffeine intake (r = 0.060, p = 0.490) or having read/heard about osteoporosis (r = -0.093, p = 0.283). For health belief scores, significant negative correlations were found with exposure to sun (r = -0.391, p < 0.0001) and nutrient-rich calcium intake (r = -0.265, p = 0.002), suggesting that higher sun exposure or lower calcium intake may be associated with fewer osteoporosis-related health behaviors. Conversely, a moderate positive correlation was observed between health belief scores and caffeine intake (r = 0.193, p = 0.025), indicating that healthier behaviors may be linked to moderate caffeine consumption. No significant relationship was found between health belief scores and regular exercise (r = 0.111, p = 0.198), although a slight positive trend was noted. These findings underscore the complex interactions between osteoporosis knowledge, lifestyle factors, and health behaviors in preventing the condition.

A regression analysis was conducted to examine the impact of age, gender, academic year, and prior osteoporosis awareness on osteoporosis knowledge and health belief scores. For osteoporosis knowledge, the overall model was not statistically significant, F(4,131) = 1.638, p = .169, explaining 4.8% (R² = .048) of the variance in osteoporosis knowledge. Among the predictors, only academic year was statistically significant (B = 1.874, p = .027), suggesting that students in later academic years had higher osteoporosis knowledge. Other variables, including age (p = .467), gender (p = .820), and prior osteoporosis awareness (p = .230), did not show significant associations with osteoporosis knowledge. For health belief scores, the model was not statistically significant, F(5,130) = 0.414, p = .838, explaining only 1.6% (R² = .016) of the variance in health belief scores. None of the individual predictors reached statistical significance (p > .05), suggesting that these variables do not significantly influence health belief about osteoporosis, as presented in Table 4.

**Table 4:** Factors Influencing Osteoporosis Knowledge and Health Belief Scores.

Predictor Variable	B	Std. Error	Beta	t	Sig.	95% CI (Lower–Upper)
**Osteoporosis Knowledge**						
Constant	3.182	11.530	–	0.276	0.783	-19.634–25.998
Age	0.391	0.536	0.065	0.730	0.467	-0.668–1.450
Gender	-0.180	0.787	-0.020	-0.228	0.820	-1.739–1.379
Academic Year	1.874	0.840	0.204	2.230	0.027	0.211–3.536
Osteoporosis Awareness	-2.755	2.284	-0.104	-1.206	0.230	-7.273–1.763
**Health Belief Scores**						
Constant	100.413	61.941	–	1.621	0.107	-22.122–222.947
Age	0.711	2.964	0.022	0.240	0.811	-5.153–6.574
Gender	2.958	4.284	0.061	0.690	0.491	-5.517–11.432
Academic Year	2.837	4.555	0.057	0.623	0.534	-6.174–11.848
Osteoporosis Knowledge	0.123	0.480	0.023	0.256	0.798	-0.826–1.072

## Discussion

To our knowledge, this is the first study to evaluate osteoporosis knowledge and beliefs among maternity nursing students in Palestine. While numerous worldwide studies have examined these factors in college students, they predominantly focus on females due to the fact that females face a higher risk of developing osteoporosis.

Osteoporosis is a global public health concern, primarily affecting postmenopausal women. University students, being the youngest part of society, can benefit from knowledge about osteoporosis, especially for nursing students. This knowledge can inform healthier lifestyles and nutrition choices to prevent bone thinning. Additionally, well-informed maternity nursing students play a vital role in raising community awareness about osteoporosis.

The present study revealed that maternity nursing students had an average level of knowledge about osteoporosis, with a mean score of 10.38 (SD = 4.48), which aligns with similar scores among Malaysian medical science students, including nurses [33]. A consistent finding of moderate knowledge was also observed among American college students and Saudi Arabian university students [23,34]. In contrast, inadequate knowledge about osteoporosis was reported among university students, including nursing students, in Jordan, Syria, Pakistan, and Saudi Arabia, as well as among college students in China [24-27,35,36]. However, a notable exception is a Saudi Arabian study that reported even higher levels of knowledge about osteoporosis among university students. This was attributed to the presence of an osteoporosis awareness and prevention campaign facilitated through the Center of Excellence for Osteoporosis at King Abdulaziz University. These collective findings emphasize the importance of providing comprehensive osteoporosis education for both nursing and university students.

In addition, the results demonstrated a noteworthy disparity in osteoporosis knowledge scores between fourth-year and third-year nursing students. Specifically, fourth-year students exhibited a significantly higher mean score of 11.4 out of a possible 20 points, in contrast to third-year students who scored 9.74. Consistently, previous studies reported that senior students scored better in osteoporosis knowledge compared to junior students [33,37]. The observed discrepancy in osteoporosis knowledge between the two groups can be attributed to the fact that fourth-year nursing students have completed comprehensive medical-surgical and geriatric nursing courses, while third-year students have only completed medical-surgical nursing I and are just beginning medical-surgical nursing II, during which osteoporosis is introduced in their curriculum.

Understanding risk factors and preventive measures is essential in preventing or delaying the onset of osteoporosis and its associated health issues. This knowledge is invaluable for healthcare policymakers and facility nurses in implementing effective prevention programs. While most respondents in this study recognized that osteoporosis increases the risk of bone fractures, only 5.9% of students mistakenly believed that osteoporosis causes symptoms before a fracture occurs. This highlights a critical gap in knowledge, as osteoporosis is often asymptomatic until a fracture happens, reinforcing the need for education on risk factor identification and early screening. Maternity nursing students did not uniformly recognize it as primarily affecting women; only 44.9% knew it was not more prevalent in men. Knowledge of risk factors varied from low to average. Participants struggled to identify race, postmenopausal status, salty diet, and alcohol intake as potential risks but correctly identified age, smoking, and family history. These findings align with prior studies showing insufficient awareness of risk factors [24,25,35].

Regarding health beliefs, 72.1% of the participants in this study displayed a positive belief toward learning about osteoporosis and adhering to recommendations. However, they perceived themselves as at lower risk for developing osteoporosis. Our study found a moderately low perceived susceptibility (mean score: 13.26/30). Similar studies in the USA also reported low susceptibility among university and college students. Ford et al. reported a mean score of 13.4/30 (43%) for university students [36], and Edmonds et al. found 13.6/30 (45%) for college students [34]. Likewise, studies in China, Pakistan, Malaysia, Saudi Arabia, Syria, and Jordan consistently reported low susceptibility scores. The observed low to moderate susceptibility may be due to young adults perceiving osteoporosis as a disease primarily affecting older individuals, its often-asymptomatic nature, and potentially inadequate knowledge about it. Additionally, they might link osteoporosis mainly with elderly women over seventy. People tend not to see themselves at risk until they experience symptoms.

Maternity nursing students see themselves as moderately susceptible to osteoporosis, but 61.3% believe it’s a serious disease that could significantly impact their lives. This aligns with earlier findings that university students rate osteoporosis severity moderately (mean score: 17.73/30), higher than susceptibility. Similar results were seen in U.S. college students and Damascus nursing students [25,34]. However, some studies showed lower seriousness scores in U.S. and Chinese college students [36]. In contrast, a recent study found medical students in Malaysia rated seriousness notably higher (20/30). This study’s results align with most prior research, indicating young adults generally perceive osteoporosis with moderate seriousness. It suggests diverse university student groups, including nursing, may have varied perceptions based on their geographic locations, educational backgrounds, or training environments.

While 72% of maternity nursing students recognize the benefits of exercise for osteoporosis, 52% perceive moderately low barriers. Similarly, 66.3% understand the benefits of calcium intake, but 60% perceive moderately high barriers. Despite positive views on exercise and calcium intake, they face significant barriers, indicating negative health beliefs. Common barriers to exercise include regular exercise being a challenge since it would be a new habit, feeling physically unprepared, family discouragement, and lack of a suitable place to exercise. Likewise, Sayed-Hassan et al. found similar results, with approximately 25% citing family discouragement and a lack of suitable places to exercise as barriers [25]. These findings align with studies by Althobiti et al. in Saudi Arabia [26] and Ford et al. in the USA and China [36]. In contrast, Edmonds et al. reported fewer college students citing family discouragement as a barrier to exercise [34].

Maternity nursing students, like their counterparts in the USA, Saudi Arabia, Jordan, and Syria, recognize the significant benefits of calcium intake for osteoporosis [24,26,33,34,36]. The findings confirm that maternity nursing students know and understand the benefits of calcium to bone health. This may be because most of them had been exposed to information related to osteoporosis and calcium-rich foods and were aware of the benefits of calcium. However, they are natural in their belief regarding the barriers to calcium intake. This aligns with studies by Sayed-Hassan et al. [25] and Chiang [33], showing medical science and nursing students acknowledging barriers, though less so than US college students [34]. Conversely, college students in the USA, China, Saudi Arabia, and Jordan reported fewer barriers to calcium intake [24,26,36]. Interestingly, participants in this study expressed higher confidence in the benefits of calcium intake compared to exercise, indicating a greater readiness to change dietary habits than exercise-related behaviors.

It is worth noting that despite the availability of information about specific risk factors, there appears to be a disconnect between this information and the actual lifestyles of the participants. Many of them reported consuming caffeine, leading sedentary lives, and having a low calcium intake. Interestingly, there are regional similarities in lifestyle practices among participants from Jordan [24] and Saudi Arabia [26], which differ from the habits of their Chinese counterparts [10]. This suggests that regional factors may play a significant role in shaping lifestyle choices.

The health motivation subscale assessed beliefs regarding learning about health issues and staying healthy. Maternity nursing students in this study showed a strong sense of personal responsibility for their well-being, as reflected in their moderately high health motivation score (21.40), representing 69% of the maximum achievable score. Consistently, Ford et al. found similar findings among US and Chinese college students [36]. As well as medical students in Malaysia, orthopedic nurses in China also demonstrated high health motivation [32,33]. In contrast, some studies reported more neutral beliefs about health motivation, particularly among students in the USA, Saudi Arabia, and nursing students in Syria [25,26,34]. This high motivation may be attributed to the comprehensive education and training nursing students receive in health-related subjects, emphasizing health promotion and prevention. These factors contribute to their strong commitment to maintaining a healthy lifestyle.

This strong health motivation among maternity nursing students presents an opportunity to enhance osteoporosis education and prevention efforts. The findings of this study highlight critical areas for enhancing osteoporosis education and prevention programs among maternity nursing students. The moderate knowledge levels observed suggest a need for integrating more comprehensive osteoporosis-related content into nursing curricula, particularly focusing on risk factors, early symptoms, and preventive strategies. Since fourth-year students demonstrated higher knowledge levels, incorporating osteoporosis education earlier in their training could improve awareness among junior students. Additionally, the low perceived susceptibility among participants underscores the importance of targeted awareness campaigns emphasizing that osteoporosis is not solely an elderly disease but can be influenced by early lifestyle choices. Addressing barriers to exercise and calcium intake through structured interventions, such as promoting accessible exercise programs and calcium-rich dietary options, could help improve health behaviors. Finally, leveraging the strong health motivation among nursing students can enhance their role as advocates for osteoporosis prevention, equipping them to educate patients and communities effectively.

### Limitations of the study

Although this study provides valuable insights, some limitations are acknowledged. The generalizability of this study’s findings is limited due to several factors. The use of convenience sampling from a single university in Palestine restricts the results’ applicability to other regions, institutions, or cultural contexts. The relatively small sample size may not fully represent the diversity of the student population, limiting broader extrapolation. Additionally, the specific cultural and healthcare environment in which the study was conducted may influence osteoporosis knowledge and health beliefs, which could differ elsewhere. To improve generalizability, future studies should include larger, more diverse samples across multiple universities and countries, considering variations in education, culture, and healthcare practices, to better inform global public health strategies. Additionally, the True/False format of the OKAT may have encouraged guessing, which could affect the accuracy of the knowledge assessment. Alternative assessment methods, such as multiple-choice or open-ended questions, could improve accuracy. Furthermore, examining the effectiveness of osteoporosis education programs and prevention strategies across different cultural contexts would be valuable. Longitudinal studies could offer insights into how knowledge and beliefs evolve over time, especially as students’ progress through their academic careers and gain more clinical experience. The study focused solely on knowledge’s impact on preventive behaviors regarding osteoporosis, but human behavior is influenced by various factors, such as cultural, psychological, and environmental elements, which were not explored. Future research should consider these factors for a more comprehensive understanding of preventive behaviors.

## Conclusion

The study reveals that maternity nursing students have moderate knowledge of osteoporosis but low perceived susceptibility, with many failing to recognize key risk factors and preventive measures. This gap between awareness and lifestyle choices, including inadequate calcium intake and limited physical activity, emphasizes the need for more comprehensive osteoporosis education within nursing curricula. Given that nursing students are poised to play a pivotal role in promoting public health, increasing awareness and knowledge about osteoporosis prevention is critical. By integrating osteoporosis-related content into academic programs and encouraging preventive behaviors, educators can empower future healthcare professionals to improve bone health, particularly during the key period of peak bone mass development. Moreover, this effort can foster a broader cultural shift toward proactive health management, ultimately contributing to reduced osteoporosis risk across generations.

## Supporting information

S1 Table Detailed scores for different knowledge items. (DOCX)
