# Supplementary material for: Understanding osteoporosis knowledge and health beliefs in maternity nursing students: a cross-sectional study
Source: PLoS One. 2025 May 19;20(5):e0323851. doi: 10.1371/journal.pone.0323851 (PMC12088013; doi:10.1371/journal.pone.0323851)
Supplement: S1 Table — (DOCX) [file pone.0323851.s001.docx]

Detailed scores for different knowledge items

| Knowledge of osteoporosis among maternity nursing students (N=136) | | |
| --- | --- | --- |
| Questions | True % (n) | False % (n) |
| 1. Osteoporosis leads to an increased risk of bone fractures. | 94.1 (128) ^*^ | 5.9 (8) |
| 1. Osteoporosis usually causes symptoms before a fracture occurs. | 94.1 (128) | 5.9 (8) ^*^ |
| 1. Having a higher peak bone mass at the end of childhood gives no protection against the development of osteoporosis in later life. | 56.4 (77) | 43.4 (59) ^*^ |
| 1. Osteoporosis is more common in men. | 55.1 (75) | 44.9 (61) ^*^ |
| 1. Cigarette smoking can contribute to osteoporosis. | 71.3 (97) ^*^ | 28.7 (39) |
| 1. White women are at the highest risk of fracture as compared to other races. | 29.4 (40) ^*^ | 70.6 (96) |
| 1. A fall is just as important as low bone strength in causing fractures. | 56.6 (77) ^*^ | 43.4 (59) |
| 1. By age 80, the majority of women have osteoporosis. | 64.7 (88) ^*^ | 35.3 (48) |
| 1. From age 50, most women can expect at least one fracture before they die. | 52.2 (71) ^*^ | 47.8 (65) |
| 1. Any type of physical activity is beneficial for osteoporosis. | 78.7 (107) | 21.3 (29) ^*^ |
| 1. It is easy to tell whether I am at risk of osteoporosis by my clinical risk factors. | 76.5 (104) ^*^ | 23.5 (32) |
| 1. Family history of osteoporosis strongly predisposes a person to osteoporosis. | 68.4 (93) ^*^ | 31.6 (43) |
| 1. Adequate calcium intake can be achieved from two glasses of milk a day. | 66.2 (90) ^*^ | 33.8 (46) |
| 1. Sardines and broccoli are good sources of calcium for people who cannot take dairy products. | 61.8 (84) ^*^ | 38.2 (52) |
| 1. Calcium supplements alone can prevent bone loss. | 55.1 (75) | 44.9 (61) ^*^ |
| 1. Alcohol in moderation has little effect on osteoporosis. | 36.8 (50) ^*^ | 63.2 (86) |
| 1. High salt intake is a risk factor for osteoporosis. | 47.1 (64) ^*^ | 52.9 (15) |
| 1. There is a small amount of bone loss in the ten years following the onset of menopause. | 80.9 (110) | 19.1 (26) ^*^ |
| 1. Hormone therapy prevents further bone loss at any age after menopause. | 38.2 (52) ^*^ | 61.8 (84) |
| 1. There are no effective treatments for osteoporosis available in Australia | 60.3 (32) | 39.7 (54) ^*^ |
| Note: * = Correct answer, SD = standard deviation. | | |
